# Implementation of stroke teams and simulation training shortened process times in a regional stroke network—A network-wide prospective trial

**DOI:** 10.1371/journal.pone.0188231

**Published:** 2017-12-05

**Authors:** Damla Tahtali, Ferdinand Bohmann, Natalia Kurka, Peter Rostek, Anelia Todorova-Rudolph, Martin Buchkremer, Mario Abruscato, Ann-Kathrin Hartmetz, Andrea Kuhlmann, Christian Henke, André Stegemann, Sanjay Menon, Björn Misselwitz, Anke Reihs, Stefan Weidauer, Sven Thonke, Uta Meyding-Lamadé, Oliver Singer, Helmuth Steinmetz, Waltraud Pfeilschifter

**Affiliations:** 1 Department of Neurology, University Hospital Frankfurt, Frankfurt am Main, Germany; 2 NICU Nursing Staff, University Hospital Frankfurt, Frankfurt am Main, Germany; 3 Department of Neurology, Vitos Weil-Lahn, Weilmünster, Germany; 4 Department of Neurology, Klinikum Hanau, Hanau, Germany; 5 Department of Neurology, Krankenhaus Nordwest, Frankfurt am Main, Germany; 6 Department of Neurology, Helios HSK Wiesbaden, Wiesbaden, Germany; 7 Department of Neurology, Sankt Katharinen-Krankenhaus, Frankfurt am Main, Germany; 8 Department of Neurology, Klinikum Aschaffenburg-Alzenau, Aschaffenburg, Germany; 9 Geschäftsstelle Qualitätssicherung Hessen (GQH), Eschborn, Frankfurt, Germany; University of Münster, GERMANY

## Abstract

**Background:**

To meet the requirements imposed by the time-dependency of acute stroke therapies, it is necessary 1) to initiate structural and cultural changes in the breadth of stroke-ready hospitals and 2) to find new ways to train the personnel treating patients with acute stroke. We aimed to implement and validate a composite intervention of a stroke team algorithm and simulation-based stroke team training as an effective quality initiative in our regional interdisciplinary neurovascular network consisting of 7 stroke units.

**Methods:**

We recorded door-to-needle times of all consecutive stroke patients receiving thrombolysis at seven stroke units for 3 months before and after a 2 month intervention which included setting up a team-based stroke workflow at each stroke unit, a train-the-trainer seminar for stroke team simulation training and a stroke team simulation training session at each hospital as well as a recommendation to take up regular stroke team trainings.

**Results:**

The intervention reduced the network-wide median door-to-needle time by 12 minutes from 43,0 (IQR 29,8–60,0, n = 122) to 31,0 (IQR 24,0–42,0, n = 112) minutes (p < 0.001) and substantially increased the share of patients receiving thrombolysis within 30 minutes of hospital arrival from 41.5% to 59.6% (p < 0.001). Stroke team training participants stated a significant increase in knowledge on the topic of acute stroke care and in the perception of patient safety. The overall course concept was regarded as highly useful by most participants from different professional backgrounds.

**Conclusions:**

The composite intervention of a binding team-based algorithm and stroke team simulation training showed to be well-transferable in our regional stroke network. We provide suggestions and materials for similar campaigns in other stroke networks.

## Introduction

The time interval from symptom onset to vessel recanalization is the decisive factor for good patient outcomes in acute stroke care [1,2]. Recanalizing therapies are still underutilized, even in regions with comprehensive health care systems [3]. State-of-the-art acute stroke care in every stroke ready hospital should warrant rapid clinical and radiological evaluation to confirm the suspicion of stroke and rule out contraindications to administer thrombolysis as fast as possible [4]. Just as importantly, the acute stroke algorithm should include rigorous screening for large vessel occlusion (LVO) in patients with persistent relevant stroke symptoms and a well-organized referral system to a comprehensive stroke center with capacities for endovascular stroke therapies [5].

What are the main obstacles to a widespread improvement in acute stroke care? The basic prerequisites to perform thrombolysis and identify candidates for thrombectomy are neither very complex nor costly and are available at many community hospitals. We assume that the crucial factors for excellent acute stroke care are ‘soft’ factors: a binding agreement on interdisciplinary team-work among the medical departments and nursing staff of the hospital, transmission of the relevant knowledge and skills to all personnel involved in acute stroke care supported by training to function efficiently as a team. While different approaches to streamline the acute stroke workflow and cut down on periprocedural times have been described [6,7], there is yet a scarcity of reports on how to enable junior doctors and nurses that are being trained to treat acute stroke patients to acquire the required non-technical skills (e.g. communication, decision making, management of stress). As comprehensive registries such as the United States’ GWTG programme [8] or the European SITS-MOST programme [9] still report overly long door-to-needle intervals, there is clearly an unmet need to improve hospitals’ performance in the basic procedures of acute stroke care.

Crew resource management (CRM) is a widely used concept to advance non-technical skills in high-fidelity work environments. The basis of CRM are teamwork with defined roles and tasks for each team member, situation awareness, task management and decision making [10]. We propose that a defined stroke team and a binding algorithm are a very useful strategy to transfer these capacities to acute stroke care [11]. We recently reported our single-center experience in setting up a stroke team and implementing regular simulation-based team training [11], which significantly reduced door-to-needle times as the benchmark parameter for acute stroke care and improved staff satisfaction. The aim of this quality campaign was to spread the concept of team-based acute stroke care and CRM to a regional stroke network with the help of simulation-based team-training. We found positive effects on the network-wide mean door-to-needle time and on staff satisfaction. We aim to advocate the idea of team training in acute stroke care and provide material to reproduce our course concept [12].

## Methods

### Participating hospitals

This prospective interventional study was conducted from January 2015 to December 2015 in the interdisciplinary neurovascular network of the Rhein-Main area in Germany (INVN Rhein-Main), one of 16 regional stroke networks endorsed by the German Stroke Society (DSG) which at the time consisted of 3 comprehensive and 4 regional stroke units Table 1 and has meanwhile expanded to 13 DSG-certified stroke units [13]. All stroke units of the network agreed to participate in the study, comply to the network-wide reporting standards and comply to all study activities (shaping of a hospital-specific stroke team algorithm, participation in the train-the-trainer seminar that conveyed principles of CRM and medical simulation training, and recording of the process times of all consecutive patients receiving i.v. thrombolysis).

**Table 1:** Charaterization of the INVN Rhein-Main regional stroke network’s hospitals.

Characterization of hospital	Number of stroke patients*	General or neuro ED	Binding acute stroke algorithm prior to study?	Binding acute stroke algorithm in the course of study?	Number and profession of participants in STROKE TEAM algorithm	Adopted regular simulation training since study?
university hospital, comprehensive stroke center	951	neuro ED	yes	yes	7 (+2) ED nurse, SU senior neurologist, SU resident, ED resident, Neuroradiology resident, radiology technician, laboratory technician, (2 emergency medical technicians)	yes
comprehensive stroke center	806	general ED, neuro resident on site 24/7	no	yes	9 ED resident, 2 ED nurses SU senior neurologist SU resident, 2 SU nurses neuroradiology resident radiology technician laboratory technician	yes
regional stroke unit	1045	general ED, neuro resident on site 24/7	yes	yes	7 (+1) ED nurse, SU nurse, SU senior neurologist, SU resident, radiology resident, radiology technician, laboratory technician, anasthesiology resident (in case of thrombectomy/critical care)	no
regional stroke unit	633	neuro ED	yes	yes	6 (+1) ED resident/SU resident, SU senior neurologist, radiology technician, radiology resident, laboratory technician, SU nurse, ED assistant (7 a.m-9 p.m.)	yes
regional stroke unit	1272	general ED, neuro resident on site 24/7	yes	yes	7 ED resident, Neuro resident, ED nurse, ED senior physician, SU/Neuro ICU senior physician, SU/Neuro ICU nurse, SU/ Neuro ICU resident	no
regional stroke unit	712	general ED, neuro resident on site 24/7	yes	yes	7 ED nurse, SU nurse, SU senior neurologist, 2 SU residents, radiology resident, radiology technician	in progress
comprehensive stroke center	1562	general ED, neuro resident on site 24/7	yes	yes	8 2 SU residents, 2 SU nurses, SU senior neurologist, radiology technician, radiology resident, ED nurse	no

Characteristics of the 7 network hospitals.

*patients with ischemic stroke, intracerebral hemorrhage and transient-ischemic attack treated in 2015, ED–emergency department

### Study design

**Fig 1:**
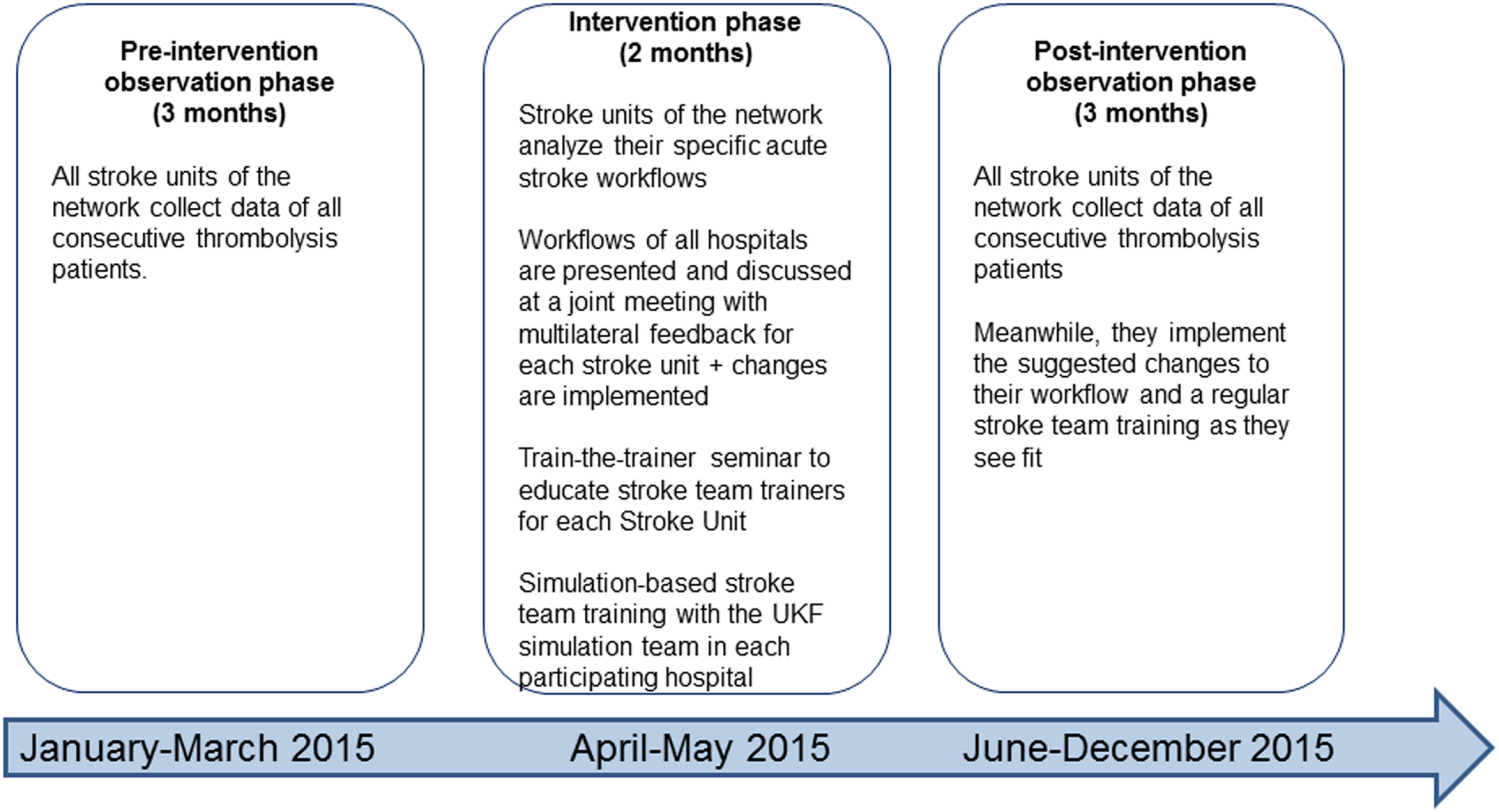
Study design and timeline of the INVN stroke team Rhein-Main quality campaign.

**Fig 2:**
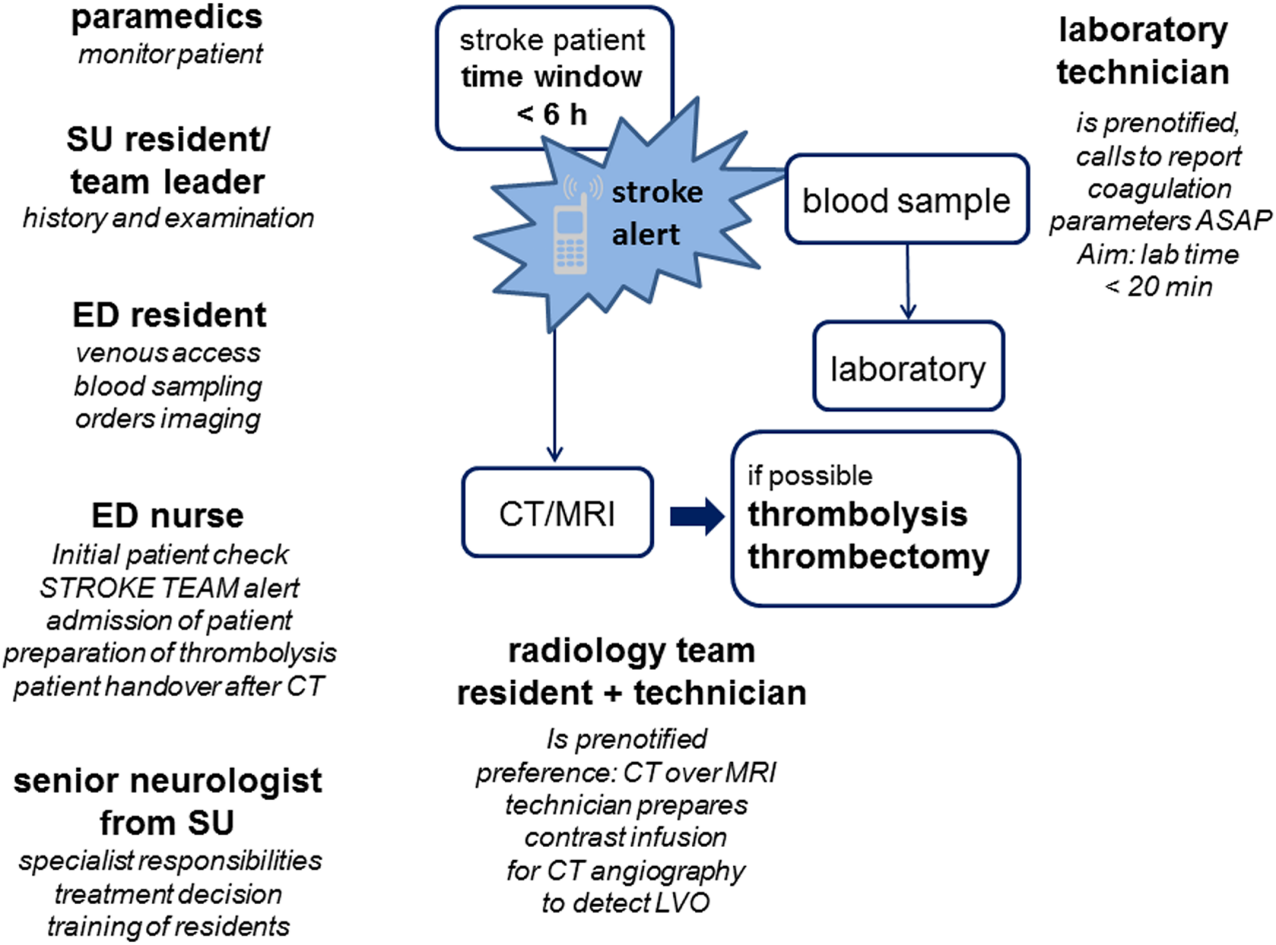
Exemplary team-based acute stroke care algorithm. The algorithm needs to be adapted to the infrastructure and staff availiability of each individual hospital. The algorithm relies on a seamless cooperation with preclinical emergency medical services and encourages working in parallel with defined tasks for each stroke team member. The stroke alert is a speed dial collective call that summons all team members simultaneously to their respective workplaces.

This interventional study comprised three phases Fig 1. During a ***3 month pre-intervention observation phase***, all consecutive thrombolysis treatments in the network were recorded. A central meeting of interdisciplinary stearing teams (3–4 persons) of each of the seven hospitals (physicians and nurses from neurology, physicians and technicians from neuroradiology) was held in which all teams described their current workflow of acute stroke care. Multilateral feedback and suggestions along the items detailed by Meretoja et al. in the description of the decade-long improvement process underlying the successful ‘Helsinki model’ supplemented by the local experience of the Frankfurt University Hospital stroke team adapted to the local circumstances at each hospital were encouraged from all participants and recorded in a structured protocol S1 File. Based on this input and the availability of personnel from the involved departments and professions at each hospital, the teams crafted hospital-specific acute stroke pathways based on a defined team with a binding set of tasks for each team member (the ‘stroke team algorithm’) that were recorded in writing during the meeting Fig 2. In the following ***2 month intervention phase***, a train-the-trainer seminar was conducted to enable stroke team trainers of the participating hospitals to set up regular stroke team training at their hospital. We conveyed basic principles of CRM and simulation training in medical education, and the participants learned debriefing techniques to facilitate experiential learning of future course participants and were given the opportunity to practice non-technical skills for successful team operations. The stroke team trainers of Frankfurt University Hospital then visited all participating hospitals once during the intervention phase to conduct an onsite 2.5 h stroke team training consisting of a theoretical course and a simulation-based team training with a high-fidelity manikin as described elsewhere [12]. The course concept and a slide-kit are available from the corresponding author upon request. During the simulation, the new stroke team algorithm was put to a test and demonstrated to all members of the participating hospital. This intervention phase was followed by a ***3 month post-intervention observation phase***, during which all consecutive thrombolysis treatments were recorded.

### Data collection

The study was approved by the institutional ethics review board of Frankfurt University Hospital (approval number 355/14). We aimed at including all consecutive adult patients who received i.v. thrombolysis at the hospitals of the network during the two observation periods. Written informed consent from from patients or their legal representatives was mandatory for data entry. To allow reliable data collection of all consecutive i.v. thrombolysis treatments in the network, we designed a minimalistic case report form consisting of a Windows Power Point document in which the treating physician pasted one image of the acute head CT to document the time of CT scanning and indicated the time of arrival and the time of thrombolysis. This document was then printed out, a hospital admission sticker rendering the time of admission, name, address and date of birth of the patients was pasted and it was transmitted to the Neurovascular Clinical Trial Unit of the University Hospital Frankfurt by fax. Median DNTs and IQR of all consecutive i.v. thrombolysis treatments before and after the intervention were compared. Statistical significance was assessed with a Mann-Whitney-U-Test.

In parallel, we retrieved data from the mandatory state-wide registry of stroke inpatient care of the federal state of Hesse (Institute of quality assurance Hesse) [14] for 2014 (the year before the intervention) and 2015 (the year during which the intervention took place). These data have an issue of imprecision because up to 2017, only time strata (< 0.5 h, 0.5–1 h, 1–2 h, etc.) for the door-to-needle time were required but the advantage of a nearly 100% completeness [14]. Therefore, we added these data as an external validation of our data collection using case report forms to compensate for eventual incomplete reporting of all thrombolysis in this context. We compared the share of acute patients receiving thrombolysis within 30 minutes after arrival in one of the INVN hospitals between 2014 and 2015 by means of Pearson’s Chi-Square-Test and analyzed the percent increase in the number of i.v. thrombolysis treatments in the hospitals of the INVN in comparison to the other stroke-ready hospitals of the state who did not participate in this intervention.

All participants (n = 104 doctors, nurses and technicians) of the onsite stroke team trainings conducted by the stroke team trainers of the Frankfurt University Hospital were asked to anonymously fill out pre-training and post-training questionnaires (S2 and S3 Files) on their perceived stroke readiness and their judgement on the practical usefulness of the teaching concept before and after the 2.5 h stroke team training session. Median ratings and IQR before and after the training were compared. Statistical significance was assessed with a Wilcoxon signed-rank test. We recorded and analyzed our data with IBM SPSS, Version 20 (IBM Corporation, Armonk, NY, USA).

## Results

### The composite intervention of setting up a binding stroke team algorithm and simulation-based stroke team training reduced door-to-needle times and increased thrombolysis rate of the stroke network

**Fig 3:**
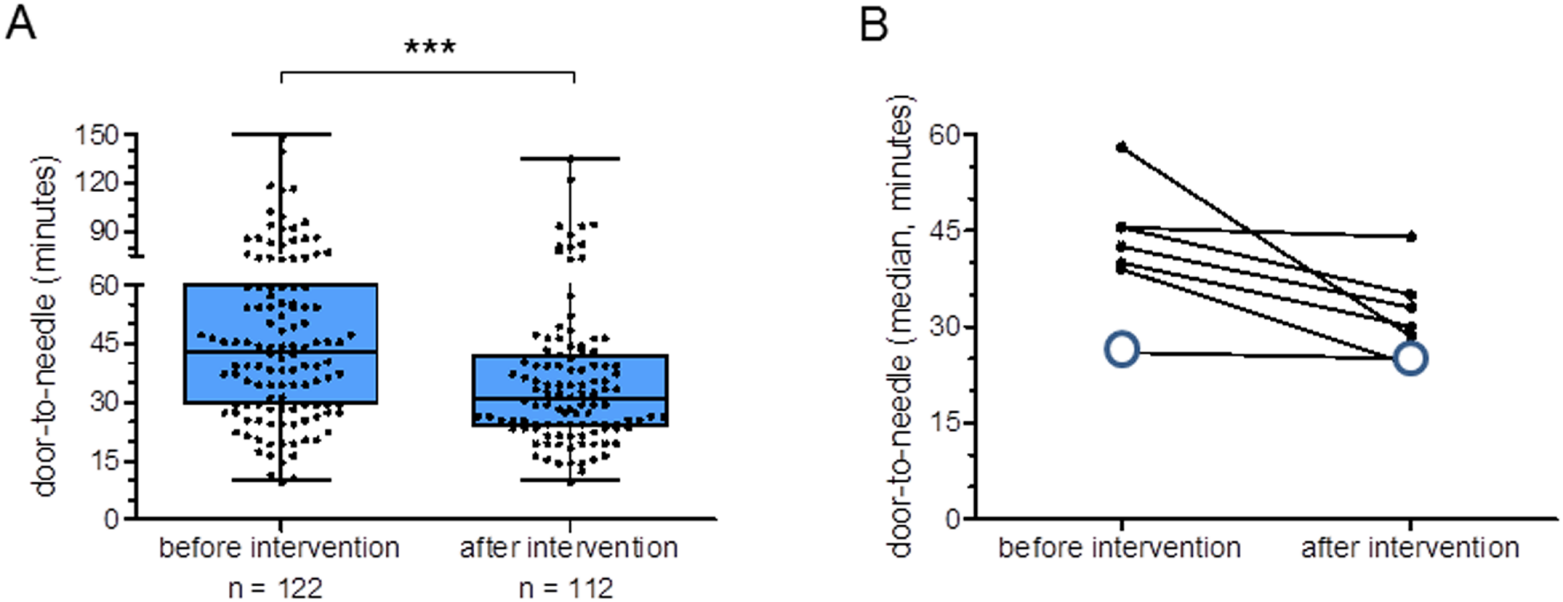
Effects of the stroke team intervention on network-wide door-to-needle times. (A) Door-to-needle times of seven stroke units of the neurovascular network before and after the composite stroke team intervention. Data are given as median, 25 to 75% interquartile range (box) and extremes (whiskers). Statistical significance was assessed with a Mann-Whitney-U test, *** p < 0.001. (B) Individual median door-to-needle times in minutes of the seven stroke units before and after the stroke team intervention. Empty circles: University Hospital Frankfurt.

In the first observation period, the network-wide median door-to-needle time was 43.0 minutes (IQR 29.8–60.0, n = 122). This time interval, which is the key benchmark parameter for acute stroke care, could be significantly shortened by 12 minutes to 31 (IQR 24.0–42.0, n = 112) minutes (p < 0.001) as recorded in the second observation period after the intervention Fig 3A. Nearly all hospitals improved their door-to-needle times during the course of the intervention. Five out of seven hospitals achieved a significant shortening of their door-to-needle times Fig 3B. At the University Hospital Frankfurt, where the expemplary algorithm Fig 2 was developed and first implemented, the fast median door-to-needle time could be maintained during the second observation period. However, the analysis of the case report forms transmitted by the six hospitals of the INVN Rhein-Main during the pre- and post-intervention observation phases did not show an increase in thrombolysis rate.

The simultaneously and independently acquired quality assurance data also showed an increase of the share of acute stroke patients receiving thrombolysis within 30 minutes after their arrival in one of the hospitals of the network from 41.5% (n = 149) in 2014 (before the intervention) to 59.5% (n = 247, p < 0.001) in 2015 (the year in which the intervention took place). In contrast to the data obtained by the case report forms of our quality campaign, the databank with mandatory data entry of all stroke patients also showed an increase in thrombolysis rate: this increase in i.v. thrombolysis 14.9% from 2014 (n = 443) to 2015 (n = 499) in the hospitals of the INVN Rhein-Main was substantially higher than the increase of the thrombolysis rate in the other stroke-ready hospitals in the state of Hesse (9.4% from n = 1479 to n = 1613).

### Efficacy of the intervention on the individual perception of stroke readiness of stroke team members in the network

**Fig 4:**
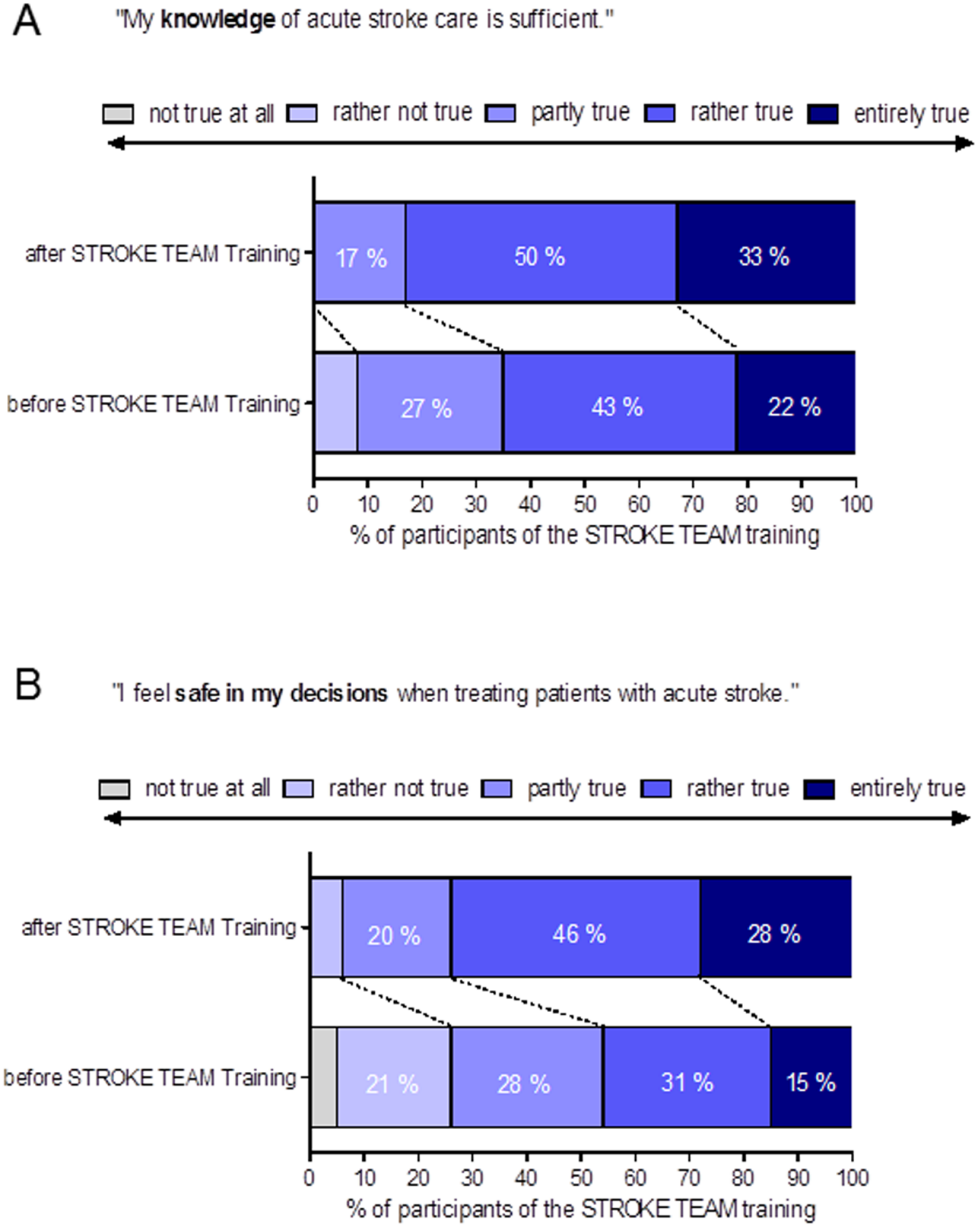
Effect of the simulation training on perceived stroke-readiness and patient safety. Responses to a questionnaire distributed to n = 152 participants of 6 stroke units (University Hospital Frankfurt did not participate actively in the intervention phase) directly before and after the simulation-based 2.5 h stroke team training. Participants were asked to respond anonymously on a 5-point scale. Statistical significance was assessed with a Wilcoxon signed-rank test. A) *** p < 0.001 and B) *** p < 0.001.

Asked by means of an anonymous questionnaire whether they had sufficient knowledge of acute stroke care at their command, one third of the participants of the on-site simulation-based trainings replied beforehand that this was “only partly true” or “rather not true”. This negative perception was improved significantly by the training, after which less than 20% still felt some degree of uncertainty Fig 4A (p < 0.001). Before the stroke team training, less than half of the participants felt safe in their decisions in acute stroke care, while almost 30% felt definitely unsecure. After the training, we observed significantly positive changes in the perception of safety with almost 70% (p < 0.001) of the stroke team members feeling that they could safely reach correct decisions in acute stroke care and less than 10% still stated a relevant degree of uncertainty Fig 4B.

### Acceptance of the stroke team training among participants in the regional stroke network

**Fig 5:**
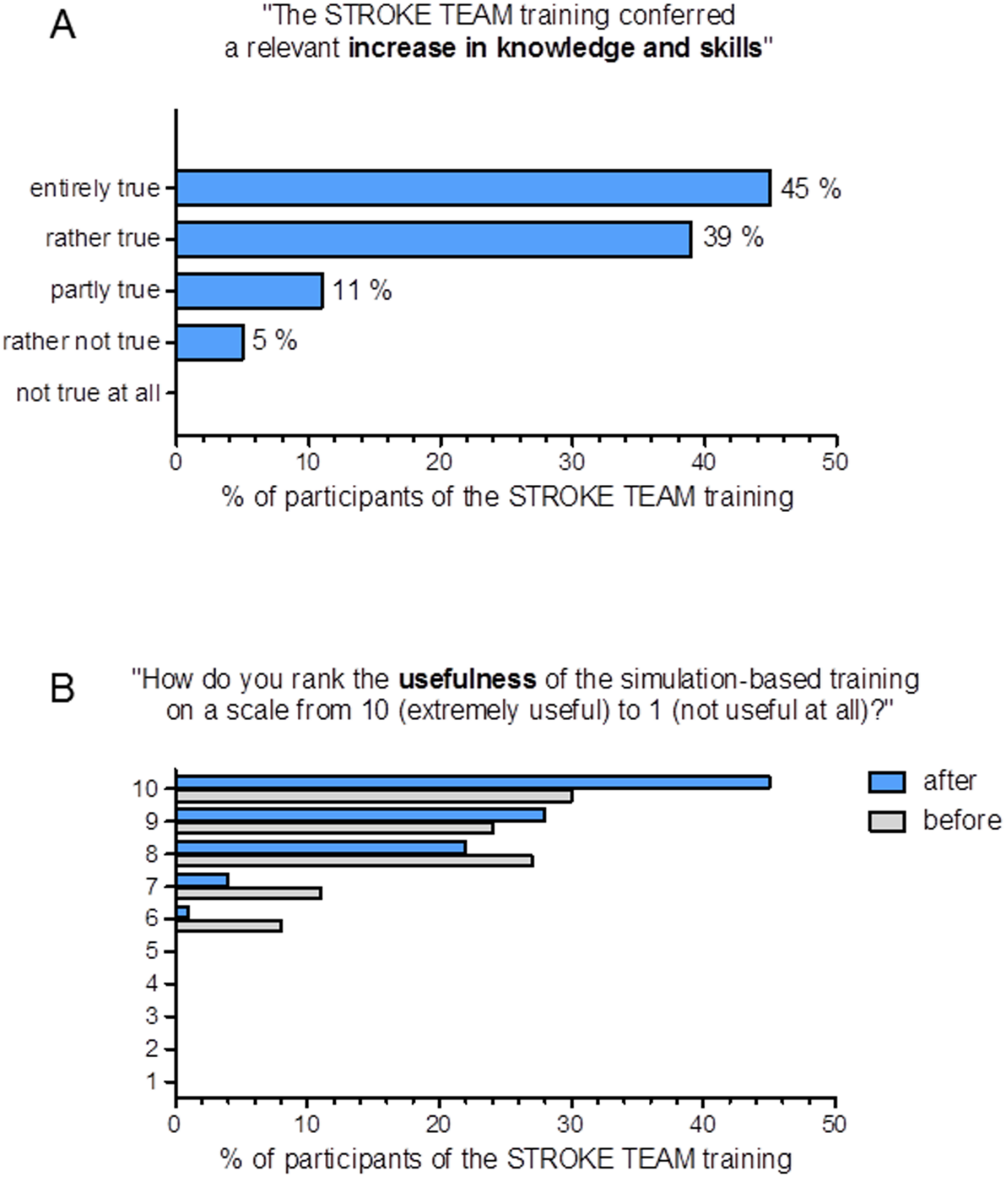
Rating of the interdisciplinary simulation training by the participants. Responses to a questionnaire distributed to n = 152 participants of 6 stroke units (University Hospital Frankfurt did not participate actively in the intervention phase) directly before and after the simulation-based 2.5 h stroke team training. Participants were asked to respond anonymously.

The participants’ answers to questions concerning the usefulness and acceptance of this new educational format in the stroke network were very positive. 84% of all participants agreed that the stroke team training conferred a relevant increase in knowledge and skills Fig 5A. The 2.5 h format consisting of a theoretical course and a practical Stroke Team simulation was presumed to be highly useful by the majority of participants (8–10 on a 1–10 scale of ‘usefulness’, 77.8%, n = 81) before the training and even more participants gave a highly positive judgement after they had completed the training (88.4%, n = 92, p < 0.001) Fig 5B. It is worth noting that despite this positive rating of course participants, so far only three of the six hospitals have taken up regular simulation training following the intervention Table 1.

## Discussion

We observed that the composite intervention of implementing a team-based algorithm to treat acute stroke patients and simulation-based team training could be successfully transferred to a regional stroke network comprising comprehensive and regional stroke centers with a significant impact on network-wide door-to-needle times, a positive effect on the thrombolysis rate and a wide acceptance among the interdisciplinary staff of the network’s stroke units. However, despite this very positive reception, so far only half of the participating hospitals that were introduced to this training concept sustained regular simulation training. Since it can be assumed that it takes regular exposure of all involved team members to CRM education to increase safety and most participants of our study wished for a repetition of the training after 6 or 12 months (data not shown), research into the hurdles faced by simulation training in clinical neurology and the development of a stroke-specific simulation curriculum would be the next logical steps.

What is the specific additional benefit of a binding stroke team algorithm and regular CRM training to all personnel involved in acute stroke care? We think that the main factor is to create a sense of shared responsibility for patient outcomes in all team members but also to foster security and reliability of the team operation acute stroke care. A review at the occasion of the 20^th^ anniversary of the first CRM trainings in anaesthesiology states that “institutionalizing patient safety as a topic of professional concern” is a key merit of CRM and recommends simulation training as a widely used and adequate vehicle to transport CRM techniques to clinical teams [15]. In the context of acute stroke, patient safety should not only be seen as avoiding adverse events such as thrombolysis-associated hemorrhage but also as achieving the maximal benefit by avoiding loss of time-to-treatment.

This project’s focus on skills and competencies clearly does not substitute medical expertise, which is achieved during the studies of medicine and the specialization for physicians or the professional education for nurses. On the contrary, one pillar of CRM is the sharing of knowledge within the team which makes the decisions of senior personnel more transparent and thus enhances the “training on the job” of junior team members and facilitates interprofessional learning.

One aim of this project was to stimulate network-wide regular simulation trainings as part of a network-wide quality culture. So far, even though the improvement program and especially the simulation trainings were viewed very positively by the training participants, only half of the hospitals have taken up regular simulation training. Currently, we sustain yearly simulation trainings centrally with a simulation team from the university hospital and try to analyze the factors that impede the implementation of local simulation trainings at the individual hospitals. Possible obstacles from our point of view are time constraints, personnel costs, lack of an adequate manikin, need for a precise course curriculum and didactic training of stroke team trainers. We hope that by analyzing these factors, we will get informative insights into the prerequisites for a stroke-specific simulation program. Simulation in medical specialty training is clearly on the rise and would certainly enrich the education in stroke medicine and neurological emergency medicine as well [16]. Therefore, the European Stroke Organisation has set up a committee to advance stroke simulation [17] that will strive to establish a stroke simulation curriculum and should aim at gathering a base of evidence for this costly and time-consuming but very effective learning method which holds the potential to enhance patient safety and the efficacy of the available stroke therapies.

We acknowledge the following limitations to our study: We did not collect outcome data, neither a short term safety endpoint such as secondary intracerebral hemorrhage at 24 h nor functional outcome at 90 days. We strongly agree that especially the short-term safety endpoint would have been important since it is a valid concern that speeding up the processes should not compromise safety. Short-term safety is a point that should not be missed in similar evaluations in the future. However, a secondary analysis of data from the ECASS-3 trial showed clearly that while the number needed to treat (NNT) to benefit one patient goes up with time from symptom onset, the NNT to the harm steeply falls, indicating that thrombolysis is safer when administered early [18]. Another concern is that we cannot discern the relative impact of each component of this complex intervention. From our point of view, the main components are ‘recording and reporting of process times’, ‘algorithm setup’, ‘awareness on the importance of team communication’ and ‘simulation-based team training’. It may well be that the multicentric observation alone lead to behavioural changes that translated into improved process times (Hawthorne effect) [19]. Nevertheless, the 2015/2016 data from the statewide quality control registry argue against a drop in performance after the completion of the campaign. Another point concerns the questionnaire-reported efficiency and acceptance of the new teaching concept. Of course, we cannot fully exclude that the favourable evaluation in the participant questionnaires was not at least partly attributable to social adequacy. However, we tried to circumvent this by anonymous answers demanding only minimal personal data from the respondants and by a central collection bypassing the participants direct supervisors.

## Summary/Conclusions

In this prospective observational study, we found that setting up a binding team-based algorithm and conducting simulation training was well-transferable to other hospitals within a regional stroke network. This composite intervention significantly improved process times and was highly accepted among participants from different professional backgrounds. The two campaign meetings with open communication of each hospitals specific workflow and multilateral feedback enhanced the climate of colleagueship and consciousness for safety and quality of care within the network. Currently, simulation training is offered centrally by the stroke simulation team of the university hospital and only half of the hospitals have taken up regular simulation training. Better knowledge of factors impeding simulation training in clinical neurology and a stroke-specific simulation program flanked by didactic education of stroke team trainers could lead to a wider use of simulation training for acute stroke.

## Supporting information

S1 File Stroke team algorithm checklist. This checklist was used to analyze options for improvement at each hospital during the train-the-trainer seminar.
(DOCX)

S2 File Pre-training questionnaire. This questionnaire was handed out to the participants before the simulation training at each hospital of the network.
(DOCX)

S3 File Post-training questionnaire. This questionnaire was handed out to the participants after the simulation training at each hospital of the network.
(DOCX)
