# Supplementary material for: Implementation of stroke teams and simulation training shortened process times in a regional stroke network—A network-wide prospective trial
Source: PLoS One. 2017 Dec 5;12(12):e0188231. doi: 10.1371/journal.pone.0188231 (PMC5716597; doi:10.1371/journal.pone.0188231)
Supplement: S1 File — This checklist was used to analyze options for improvement at each hospital during the train-the-trainer seminar. (DOCX) [file pone.0188231.s001.docx]

**Stroke Team Algorithm Checklist**

How many staff members are currently involved in your acute stroke care algorithm?

(*Indicate number and profession):*

How many staff members could you realistically involve in an acute stroke care algorithm lasting 30 min per incoming patient? (*Indicate number and profession)*

yes not yet, not feasible

but feasible

*Helsinki recommendations, adapted from Meretoja A et al., Neurology 2012*

EMS education/involvement ⎕ ⎕ ⎕

prenotification by EMS ⎕ ⎕ ⎕

pre-acquisition of history ⎕ ⎕ ⎕

alarm and pre-order of tests ⎕ ⎕ ⎕

pre-mixing of t-PA ⎕ ⎕ ⎕

direct patient transfer to CT ⎕ ⎕ ⎕

reduced imaging (CT preferred) ⎕ ⎕ ⎕

no-delay CT interpretation ⎕ ⎕ ⎕

point-of-care INR ⎕ ⎕ ⎕

delivery of t-PA on CT table ⎕ ⎕ ⎕

rapid assessment (NIHSS-focussed) ⎕ ⎕ ⎕

*additional Frankfurt recommendations, adapted from Tahtali et al., JoVE 2017*

binding team-based algorithm (stroke team) ⎕ ⎕ ⎕

simultaneous notification of team ⎕ ⎕ ⎕

well-defined tasks, working in parallel ⎕ ⎕ ⎕

perform CT + CTA for all patients ⎕ ⎕ ⎕

with relevant stroke symptoms

regular training of junior team members ⎕ ⎕ ⎕

use of simulation training ⎕ ⎕ ⎕
