# Supplementary material for: Implementation of stroke teams and simulation training shortened process times in a regional stroke network—A network-wide prospective trial
Source: PLoS One. 2017 Dec 5;12(12):e0188231. doi: 10.1371/journal.pone.0188231 (PMC5716597; doi:10.1371/journal.pone.0188231)
Supplement: S2 File — This questionnaire was handed out to the participants before the simulation training at each hospital of the network. (DOCX) [file pone.0188231.s002.docx]

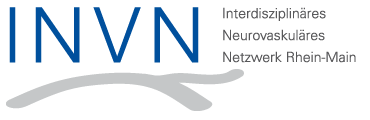


STROKE TEAM Training

*questionnaire* ***before*** *simulation*

Sex: m ☐ f ☐ profession/function: _________________________________________

Involved in acute stroke care since: 1-12 months ☐ 12-24 months ☐ >24 months ☐

**1) I have solid basic knowledge on the topic “stroke”.**

| ☐ | ☐ | ☐ | ☐ | ☐ |
| --- | --- | --- | --- | --- |
| not true at all | rather not true | partly true | rather true | entirely true |

**2) I feel safe and competent when treating patients with acute stroke.**

| ☐ | ☐ | ☐ | ☐ | ☐ |
| --- | --- | --- | --- | --- |
| not true at all | rather not true | partly true | rather true | entirely true |

**3) Please estimate the share of patients among those that reach our hospital as „stroke within time window“ actually that acutally receive iv thrombolysis.**

_________ %

**4) Please estimate our average door-to-needle time?**

_______ min

**5) Please estimate the share of errors in medicine that occur secondary to a lack of knowledge.**

________ %

**6) Please estimate the share of errors in medicine that occur secondary to poor teamwork, insufficient communication and inefficient distribution of the workload?**

________ %

**7) Please give us your expectation: On a scale from 1 (lowest) to 10 (highest) how would you rank the usefulness for you personally of the stroke team course that you are about to participate in?**

No use at all Extremely useful

1 2 3 4 5 6 7 8 9 10

**8) What do you personally expect from the simulation-based stroke team training?**

_______________________________________________________________________________

_______________________________________________________________________________
