# Supplementary material for: Implementation of stroke teams and simulation training shortened process times in a regional stroke network—A network-wide prospective trial
Source: PLoS One. 2017 Dec 5;12(12):e0188231. doi: 10.1371/journal.pone.0188231 (PMC5716597; doi:10.1371/journal.pone.0188231)
Supplement: S3 File — This questionnaire was handed out to the participants after the simulation training at each hospital of the network. (DOCX) [file pone.0188231.s003.docx]

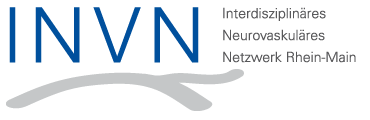


STROKE TEAM Training

*questionnaire* ***after*** *simulation*

Sex: m ☐ f ☐ profession/function: _________________________________________

Involved in acute stroke care since: 1-12 months ☐ 12-24 months ☐ months ☐

**1) I have solid basic knowledge on the topic “stroke”.**

| ☐ | ☐ | ☐ | ☐ | ☐ |
| --- | --- | --- | --- | --- |
| not true at all | rather not true | partly true | rather true | entirely true |

**2) I feel safe and competent when treating patients with acute stroke.**

| ☐ | ☐ | ☐ | ☐ | ☐ |
| --- | --- | --- | --- | --- |
| not true at all | rather not true | partly true | rather true | entirely true |

**3) The course including theory and simulation-based training significantly improved my knowledge.**

| ☐ | ☐ | ☐ | ☐ | ☐ |
| --- | --- | --- | --- | --- |
| not true at all | rather not true | partly true | rather true | entirely true |

**4) How useful was the course for you personally?**

No use at all Extremely useful

1 2 3 4 5 6 7 8 9 10

**5) How do you rate the course concept (2.5 h, theory and practical simulation training)?**

Very good Very poor

1 2 3 4 5 6 7 8 9 10

**6) What did you especially like?**

________________________________________________________________________________________________________________________________________________________________________

**7) What could be better? What was missing?**

________________________________________________________________________________________________________________________________________________________________________

**8) How do you like the fact that you could train in-situ at your actual workplace?**

Very good Very poor

1 2 3 4 5 6 7 8 9 10

**9) How realistic was the stroke simulation?**

Very good Very poor

1 2 3 4 5 6 7 8 9 10

**10) How relevant to your daily work was this course?**

Very good Very poor

1 2 3 4 5 6 7 8 9 10

**11) Do you think you can transfer the human factor concept into your daily work routine?**

Very good Very poor

1 2 3 4 5 6 7 8 9 10

**12) How often would you like a refresher of this course?**

Not necessary every 6 months yearly every two years
